# Supplementary material for: Artificial Mutations in the Nuclear Gene Encoding Mitochondrial RNA Polymerase Restore Pollen Fertility in Cytoplasmic Male Sterile Tomato
Source: Plant Biotechnol J. 2025 Oct 23;24(3):1414–27. doi: 10.1111/pbi.70417 (PMC12946495; doi:10.1111/pbi.70417)
Supplement: Supplementary file 1 — Figure S1: Bulked Segregant Analysis for the fertile and sterile population in EMS#1. Figure S2: Subcellular localization of SlRPOTm in Nicotiana benthamiana protoplasts. Figure S3: Evidence for homozygous mutation in EMS#1. Figure S4: Evaluation of the intronic mutation in EMS#1 and its effect on SlRPOTm splicing. Figure S5: Effects of heterozygous and homozygous EMS#1 mutations on vegetative tissues. Figure S6: Determination of the 5′ and 3′ UTR regions of orf137 by CR‐RT‐PCR. Figure S7: Evaluation of seed production by hand pollination. [file PBI-24-1414-s001.pptx]

## Slide 1
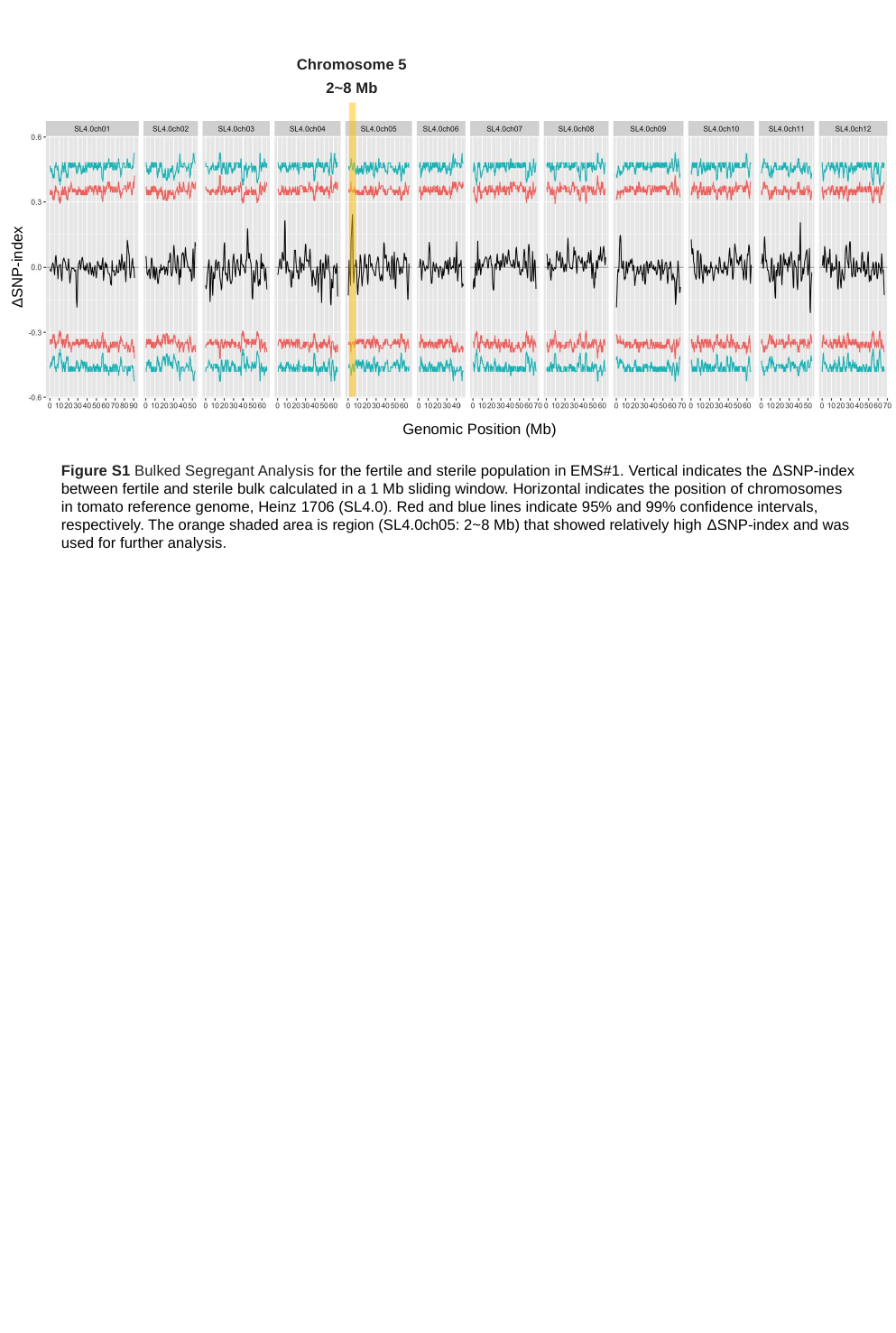

Chromosome 5 2~8 Mb
ΔSNP-index
Genomic Position (Mb)
Figure S1 Bulked Segregant Analysis for the fertile and sterile population in EMS#1. Vertical indicates the ΔSNP-index between fertile and sterile bulk calculated in a 1 Mb sliding window. Horizontal indicates the position of chromosomes in tomato reference genome, Heinz 1706 (SL4.0). Red and blue lines indicate 95% and 99% confidence intervals, respectively. The orange shaded area is region (SL4.0ch05: 2~8 Mb) that showed relatively high ΔSNP-index and was used for further analysis.

## Slide 2
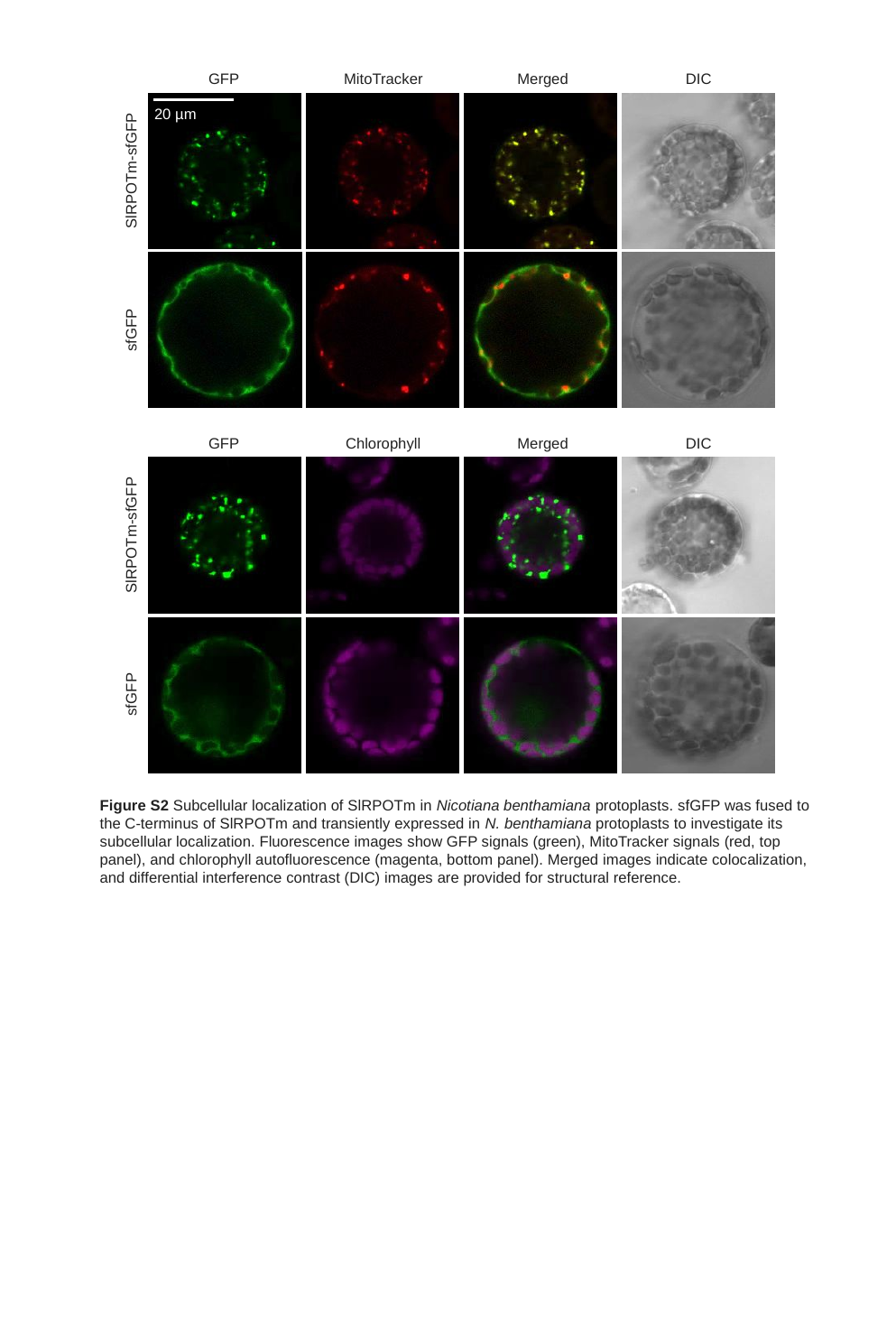

GFP
MitoTracker
Merged
DIC
20 µm
SlRPOTm-sfGFP
sfGFP
GFP
Chlorophyll
Merged
DIC
SlRPOTm-sfGFP
sfGFP
Figure S2 Subcellular localization of SlRPOTm in Nicotiana benthamiana protoplasts. sfGFP was fused to the C-terminus of SlRPOTm and transiently expressed in N. benthamiana protoplasts to investigate its subcellular localization. Fluorescence images show GFP signals (green), MitoTracker signals (red, top panel), and chlorophyll autofluorescence (magenta, bottom panel). Merged images indicate colocalization, and differential interference contrast (DIC) images are provided for structural reference.

## Slide 3
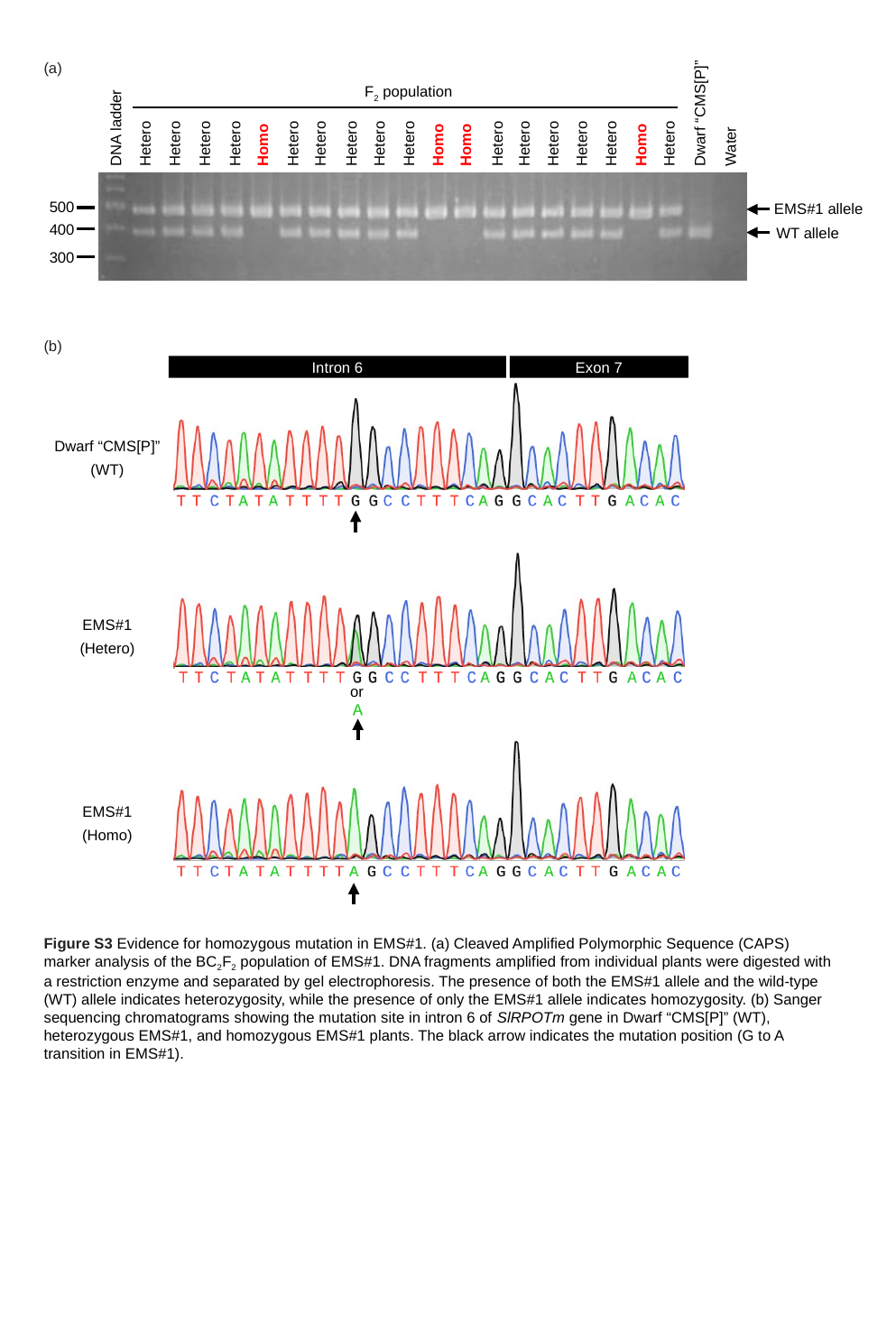

(a)
F2 population
Dwarf “CMS[P]”
DNA ladder
Water
Hetero
Hetero
Hetero
Hetero
Homo
Hetero
Hetero
Hetero
Hetero
Hetero
Homo
Homo
Hetero
Hetero
Hetero
Hetero
Hetero
Homo
Hetero
500
EMS#1 allele
400
WT allele
300
(b)
Intron 6
Exon 7
Dwarf “CMS[P]”
(WT)
EMS#1
(Hetero)
or
EMS#1
(Homo)
Figure S3 Evidence for homozygous mutation in EMS#1. (a) Cleaved Amplified Polymorphic Sequence (CAPS) marker analysis of the BC2F2 population of EMS#1. DNA fragments amplified from individual plants were digested with a restriction enzyme and separated by gel electrophoresis. The presence of both the EMS#1 allele and the wild-type (WT) allele indicates heterozygosity, while the presence of only the EMS#1 allele indicates homozygosity. (b) Sanger sequencing chromatograms showing the mutation site in intron 6 of SlRPOTm gene in Dwarf “CMS[P]” (WT), heterozygous EMS#1, and homozygous EMS#1 plants. The black arrow indicates the mutation position (G to A transition in EMS#1).

## Slide 4
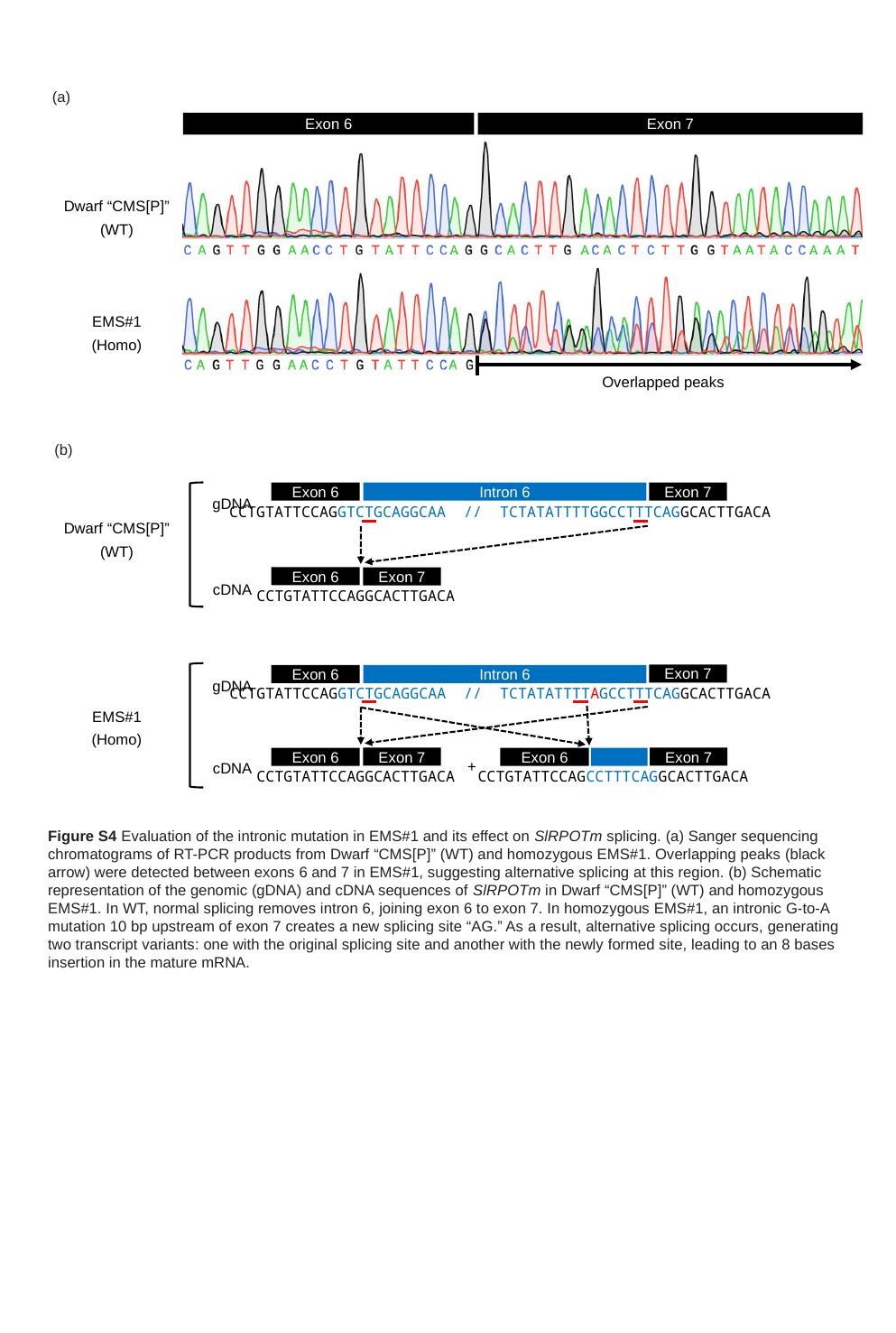

(a)
Exon 6
Exon 7
Dwarf “CMS[P]”
(WT)
EMS#1
(Homo)
Overlapped peaks
(b)
Intron 6
Exon 6
Exon 7
gDNA
CCTGTATTCCAGGTCTGCAGGCAA // TCTATATTTTGGCCTTTCAGGCACTTGACA
Dwarf “CMS[P]”
(WT)
Exon 6
Exon 7
cDNA
CCTGTATTCCAGGCACTTGACA
Exon 7
gDNA
Intron 6
Exon 6
CCTGTATTCCAGGTCTGCAGGCAA // TCTATATTTTAGCCTTTCAGGCACTTGACA
EMS#1
(Homo)
+
cDNA
Exon 7
Exon 7
Exon 6
Exon 6
CCTGTATTCCAGGCACTTGACA
CCTGTATTCCAGCCTTTCAGGCACTTGACA
Figure S4 Evaluation of the intronic mutation in EMS#1 and its effect on SlRPOTm splicing. (a) Sanger sequencing chromatograms of RT-PCR products from Dwarf “CMS[P]” (WT) and homozygous EMS#1. Overlapping peaks (black arrow) were detected between exons 6 and 7 in EMS#1, suggesting alternative splicing at this region. (b) Schematic representation of the genomic (gDNA) and cDNA sequences of SlRPOTm in Dwarf “CMS[P]” (WT) and homozygous EMS#1. In WT, normal splicing removes intron 6, joining exon 6 to exon 7. In homozygous EMS#1, an intronic G-to-A mutation 10 bp upstream of exon 7 creates a new splicing site “AG.” As a result, alternative splicing occurs, generating two transcript variants: one with the original splicing site and another with the newly formed site, leading to an 8 bases insertion in the mature mRNA.

## Slide 5
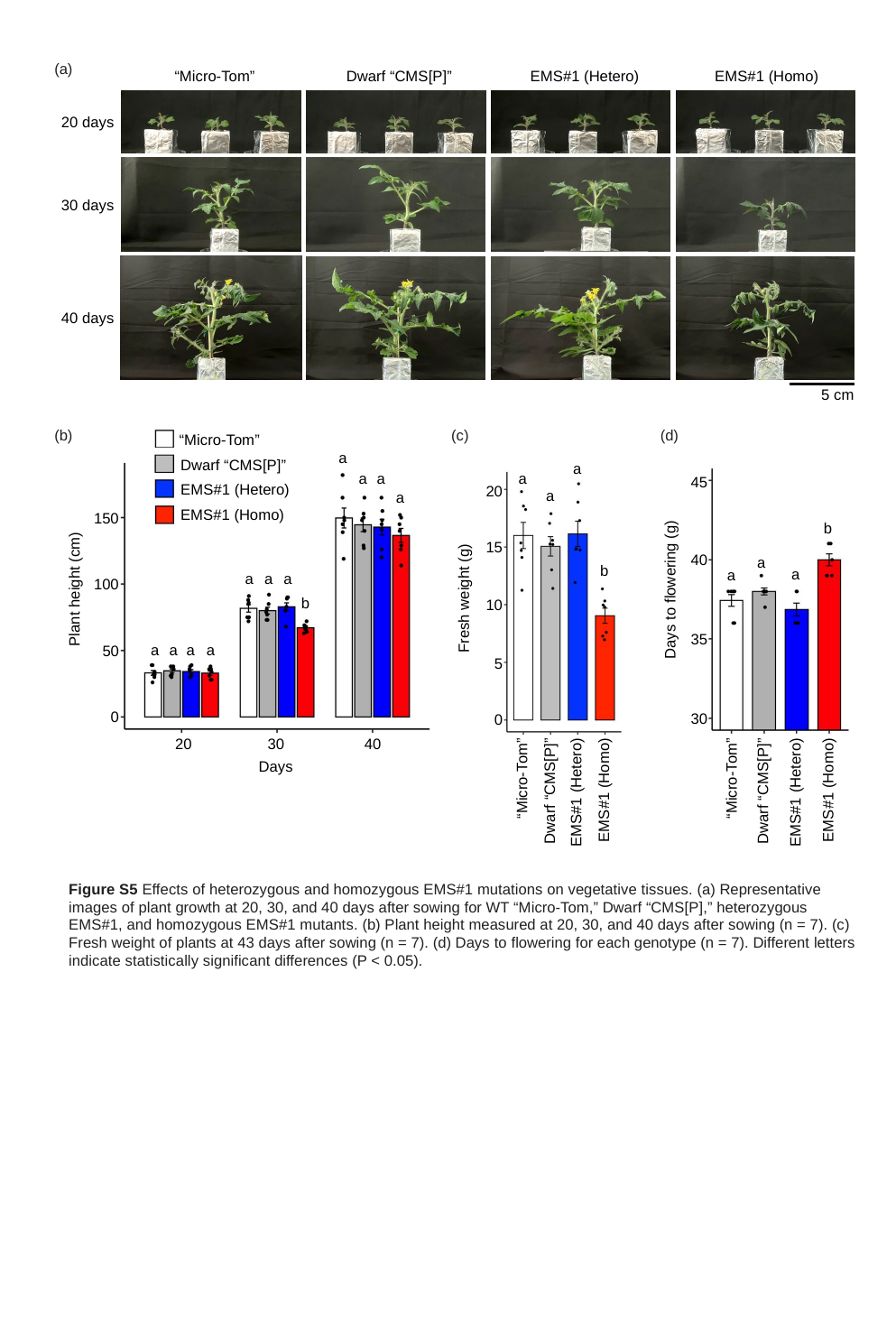

(a)
“Micro-Tom”
Dwarf “CMS[P]”
EMS#1 (Hetero)
EMS#1 (Homo)
20 days
30 days
40 days
5 cm
(b)
(c)
(d)
 “Micro-Tom”
a
Dwarf “CMS[P]”
a
a
a
a
45
EMS#1 (Hetero)
20
a
a
EMS#1 (Homo)
150
b
15
40
a
b
a
a
a
a
a
100
Plant height (cm)
Days to flowering (g)
Fresh weight (g)
b
10
35
a
a
a
a
50
5
0
30
0
20
30
40
Days
 “Micro-Tom”
 “Micro-Tom”
Dwarf “CMS[P]”
Dwarf “CMS[P]”
EMS#1 (Homo)
EMS#1 (Homo)
EMS#1 (Hetero)
EMS#1 (Hetero)
Figure S5 Effects of heterozygous and homozygous EMS#1 mutations on vegetative tissues. (a) Representative images of plant growth at 20, 30, and 40 days after sowing for WT “Micro-Tom,” Dwarf “CMS[P],” heterozygous EMS#1, and homozygous EMS#1 mutants. (b) Plant height measured at 20, 30, and 40 days after sowing (n = 7). (c) Fresh weight of plants at 43 days after sowing (n = 7). (d) Days to flowering for each genotype (n = 7). Different letters indicate statistically significant differences (P < 0.05).

## Slide 6
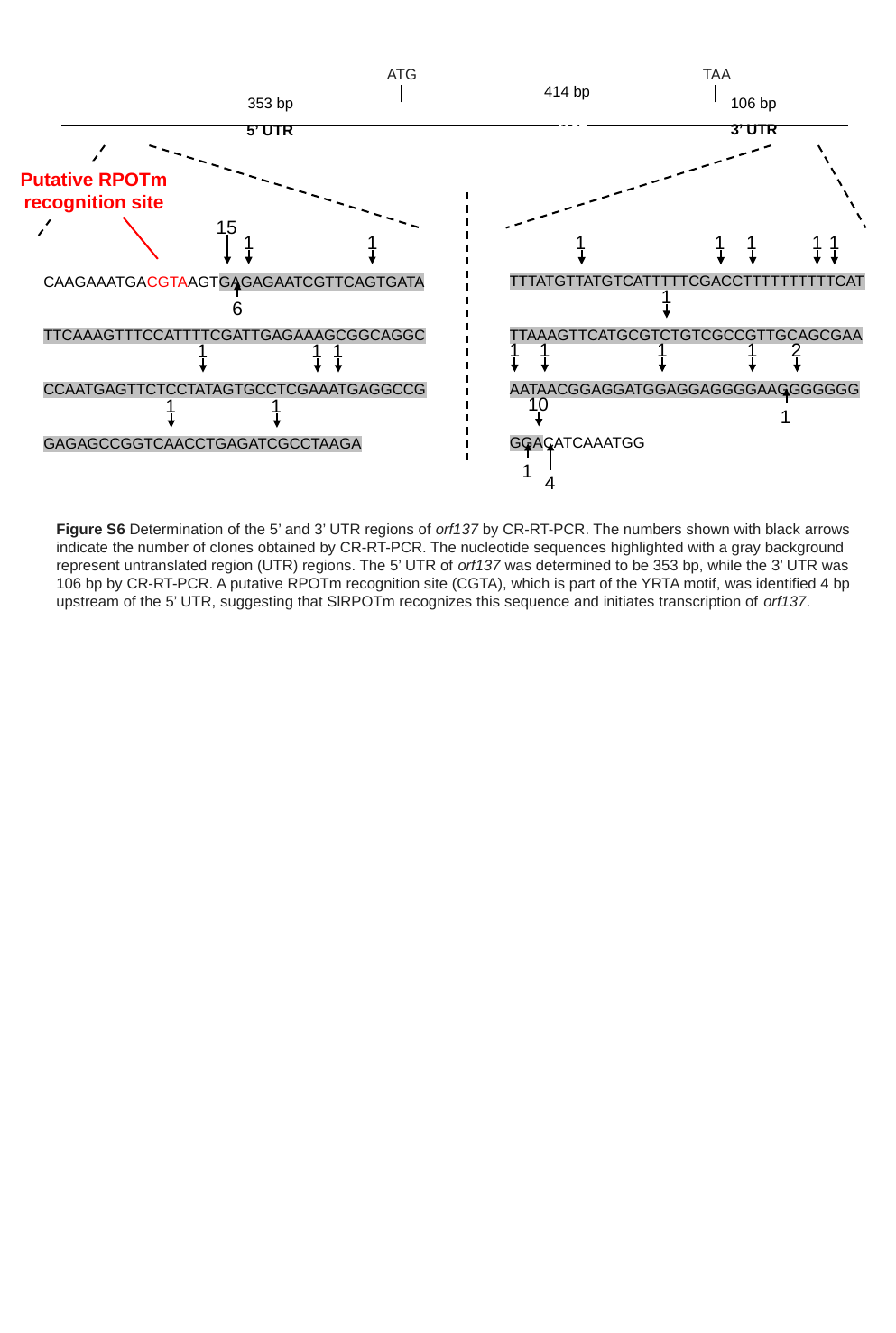

ATG
TAA
414 bp
353 bp
106 bp
3’ UTR
5’ UTR
orf137
Putative RPOTm
recognition site
15
1
1
1
1
1
1
1
TTTATGTTATGTCATTTTTCGACCTTTTTTTTTTCATTTAAAGTTCATGCGTCTGTCGCCGTTGCAGCGAAAATAACGGAGGATGGAGGAGGGGAAGGGGGGGGGACATCAAATGG
CAAGAAATGACGTAAGTGAGAGAATCGTTCAGTGATATTCAAAGTTTCCATTTTCGATTGAGAAAGCGGCAGGCCCAATGAGTTCTCCTATAGTGCCTCGAAATGAGGCCGGAGAGCCGGTCAACCTGAGATCGCCTAAGA
1
6
1
1
1
1
2
1
1
1
10
1
1
1
1
4
Figure S6 Determination of the 5’ and 3’ UTR regions of orf137 by CR-RT-PCR. The numbers shown with black arrows indicate the number of clones obtained by CR-RT-PCR. The nucleotide sequences highlighted with a gray background represent untranslated region (UTR) regions. The 5’ UTR of orf137 was determined to be 353 bp, while the 3’ UTR was 106 bp by CR-RT-PCR. A putative RPOTm recognition site (CGTA), which is part of the YRTA motif, was identified 4 bp upstream of the 5’ UTR, suggesting that SlRPOTm recognizes this sequence and initiates transcription of orf137.

## Slide 7
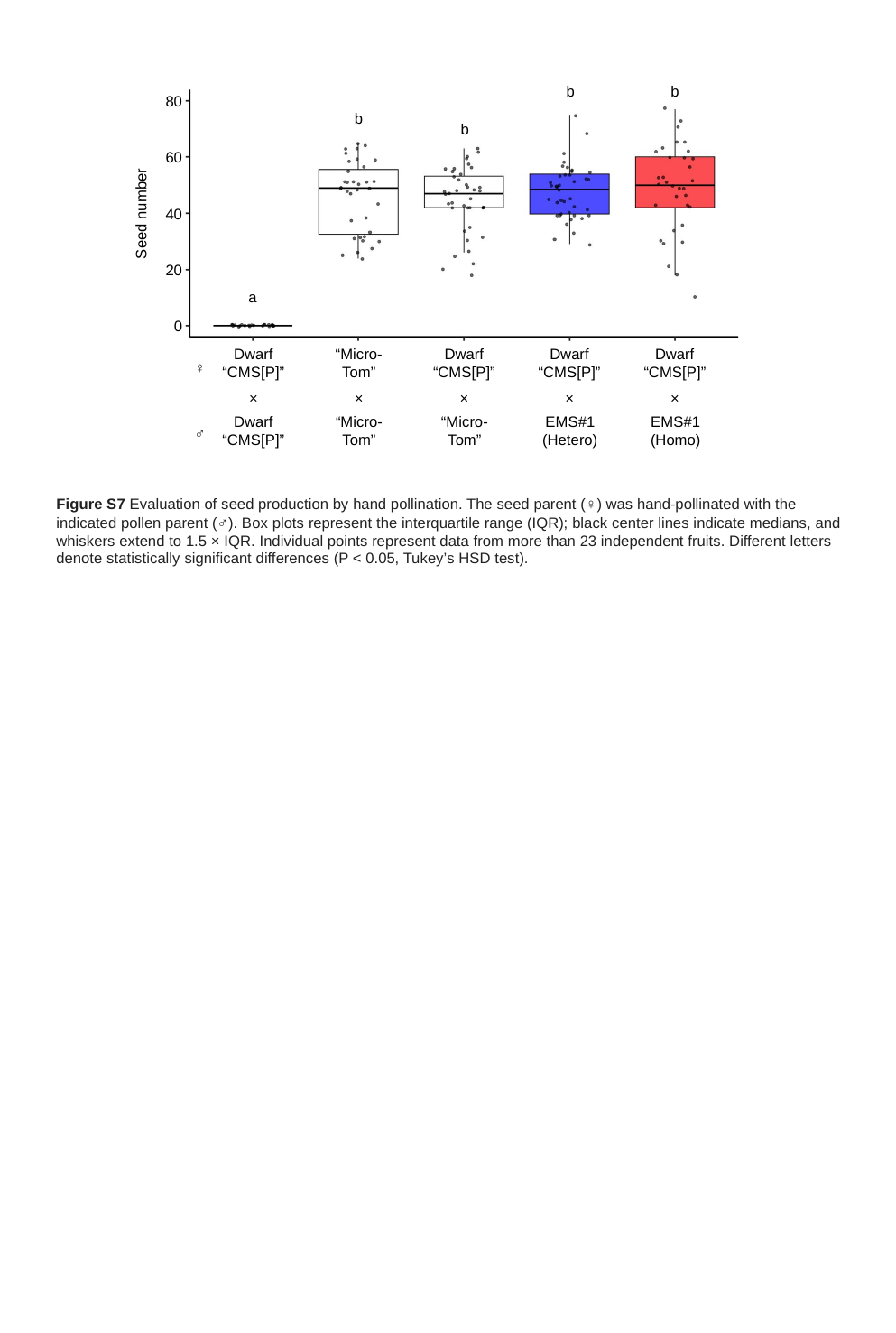

b
b
80
b
b
60
Seed number
40
20
a
0
Dwarf
“CMS[P]”
“Micro-Tom”
Dwarf
“CMS[P]”
Dwarf
“CMS[P]”
Dwarf
“CMS[P]”
♀
×
×
×
×
×
Dwarf
“CMS[P]”
“Micro-Tom”
“Micro-Tom”
EMS#1 (Hetero)
EMS#1 (Homo)
♂
Figure S7 Evaluation of seed production by hand pollination. The seed parent (♀) was hand-pollinated with the indicated pollen parent (♂). Box plots represent the interquartile range (IQR); black center lines indicate medians, and whiskers extend to 1.5 × IQR. Individual points represent data from more than 23 independent fruits. Different letters denote statistically significant differences (P < 0.05, Tukey’s HSD test).
