# Supplementary material for: Artificial Mutations in the Nuclear Gene Encoding Mitochondrial RNA Polymerase Restore Pollen Fertility in Cytoplasmic Male Sterile Tomato
Source: Plant Biotechnol J. 2025 Oct 23;24(3):1414–27. doi: 10.1111/pbi.70417 (PMC12946495; doi:10.1111/pbi.70417)
Supplement: Supplementary file 2 — Table S1: Summary of RNA‐Seq analysis. Table S2: Primers used in this study. [file PBI-24-1414-s002.pdf]

Table S1 Summary of RNA-Seq analysis.

Line	Tissue	Repeat	Total pairs	Aligned concordantly 1 time	Aligned concordantly >1 times	Overall alignment rate %
Dwarf “CMS[P]”	Anther	1	23653473	273616	125278	1.69
Dwarf “CMS[P]”	Anther	2	25135832	327827	150239	1.90
Dwarf “CMS[P]”	Anther	3	21204417	215849	98079	1.48
EMS#1 (homo)	Anther	1	21977271	145698	88268	1.06
EMS#1 (homo)	Anther	2	23193966	112544	69141	0.78
EMS#1 (homo)	Anther	3	23955898	131251	80301	0.88
Dwarf “CMS[P]”	10 min-incubated pollen	1	23214276	3613	1812	0.02
Dwarf “CMS[P]”	10 min-incubated pollen	2	22717653	11083	5940	0.07
Dwarf “CMS[P]”	10 min-incubated pollen	3	24737850	3648	1936	0.02
EMS#1 (homo)	10 min-incubated pollen	1	22676570	2895	1849	0.02
EMS#1 (homo)	10 min-incubated pollen	2	19158037	2012	1412	0.02
EMS#1 (homo)	10 min-incubated pollen	3	18896669	1918	1313	0.02
Dwarf “CMS[P]”	60 min-incubated pollen	1	23727617	3764	1929	0.02
Dwarf “CMS[P]”	60 min-incubated pollen	2	24443592	9078	5495	0.06
Dwarf “CMS[P]”	60 min-incubated pollen	3	22677189	3734	1955	0.03
EMS#1 (homo)	60 min-incubated pollen	1	25013581	2345	1593	0.02
EMS#1 (homo)	60 min-incubated pollen	2	18499882	1566	994	0.01
EMS#1 (homo)	60 min-incubated pollen	3	19679214	1401	954	0.01

Table S2 Primers used in this study.

Name	Sequence (5' to 3')	Application
EMS#1_CAPS_Fw	TCCACTAGCCCATATTCTGTCTAAT	CAPS marker for EMS#1 (PCR product is digested by HaeⅢ)
EMS#1_CAPS_Rv	CTCAGAACCATGTATGGATTGGTT	CAPS marker for EMS#1 (PCR product is digested by HaeIII)
EMS#7_CAPS_Fw	GTGAATGGGCATATGAGATATAGAGGT	CAPS marker for EMS#7 (PCR product is digested by HphⅠ)
EMS#7_CAPS_Rv	CTGATTTATGCCGTGGCATCC	CAPS marker for EMS#7 (PCR product is digested by HphⅠ)
EMS#9_CAPS_Fw	GAATTTGATCAAACTTCCTGCAGC	CAPS marker for EMS#9 (PCR product is digested by HphⅠ)
EMS#9_CAPS_Rv	CCATTGAATAAGCTGTCTTTGTTTGA	CAPS marker for EMS#9 (PCR product is digested by HphⅠ)
EMS#11_dCAPS_Fw	ACACCACCACCATATACATTTGCTAGATGTATCTTCAGCCAGCGTAAtC†	CAPS marker for EMS#11 (PCR product is digested by XbaⅠ)
EMS#11_dCAPS_Rv	ACTGCGAGAGGCATTCTCAG	CAPS marker for EMS#11 (PCR product is digested by XbaⅠ)
EMS#1_exon6_Fw	GTATGACAGAGGTGCATACTTATTTTTACCA	RT-PCR and Sanger-sequencing for an intronic mutaion in EMS#1
EMS#1_exon7_Rv	ATCTTCCCTGTCGACTAAATCAGCAA	RT-PCR and Sanger-sequencing for an intronic mutaion in EMS#1
NPTⅡ_Fw	ATGATTGAACAAGATGGATTGCAC	PCR for *NPTⅡ*
NPTⅡ_Rv	TCAGAAGAACTCGTCAAGAAGGCG	PCR for *NPTⅡ*
gRNA_Target1_Fw	ATTGACTGCGAGAGGCATTCTCAG	Construction of CRISPR-Cas9 vector
gRNA_Target1_Rv	AAACCTGAGAATGCCTCTCGCAGT	Construction of CRISPR-Cas9 vector
gRNA_Target2_Fw	ATTGCTTGGTTCTGATTTATGCCG	Construction of CRISPR-Cas9 vector
gRNA_Target2_Rv	AAACCGGCATAAATCAGAACCAAG	Construction of CRISPR-Cas9 vector
CR_sequence_Fw	GCCGTCTTGCTGATTTAGTCGA	Sequence confirmation of CRISPR-Cas9-mediated mutants
CR_sequence_Rv	ATATCCTCCACATGGTTTTCACTG	Sequence confirmation of CRISPR-Cas9-mediated mutants
orf137_RT-qPCR_Fw	GGTACATCGCTTTCTCTGTGTC	RT-qPCR for *orf137*
orf137_RT-qPCR_Rv	GAGTTTCGTCCCCGTCTTAATTTTC	RT-qPCR for *orf137*
Actin_RTqPCR_Fw	GCGAGAAATTGTCAGGGACGT	RT-qPCR for *Actin*
Actin_RTqPCR_Rv	TGCCCATCTGGGAGCTCAT	RT-qPCR for *Actin*
SlRPOTm_InFusion_Fw	GCAGGCTCCGCGGCCGCCACCATGTGGAGATACATATCAAAACAAGTTT	Amplification of *SlRPOTm* coding region for InFusion cloning
SlRPOTm_InFusion_Rv	AGCTGGGTCGGCGCGCCCGTTGAAAAAATAGGGAGATTCAAGAACT	Amplification of *SlRPOTm* coding region for InFusion cloning
pENTR/D-TOPO_InFusion_Fw	CGCGCCGACCCAGCTTTCTTGTACAAAGTT	Inverse PCR of pENTR™ /D-TOPO™ vector for InFusion cloning
pENTR/D-TOPO_InFusion_Rv	GGCCGCGGAGCCTGCTTTTTTGTACAAAGT	Inverse PCR of pENTR™ /D-TOPO™ vector for InFusion cloning
sfGFP_P1	ATGGTGAGCAAGGGCGAGGAG	Construction of pUGW_sfGFP_HSPT vector
sfGFP_P2	TTTATACAGCTCGTCCATGCCGAGAGTGATCCC	Construction of pUGW_sfGFP_HSPT vector
vector_P1	CTCCTCGCCCTTGCTCACCATCCCCATCACCACTTTGTACAAGAAAGCT	Construction of pUGW_sfGFP_HSPT vector
vector_P2	GGGATCACTCTCGGCATGGACGAGCTGTACAAGTAAGTCGACGAGCTCATATGAAGATGAAGA	Construction of pUGW_sfGFP_HSPT vector
GW_cassette_P1	GATCACAAGTTTGTACAAAAAAGC	Construction of pUGW_sfGFP_HSPT vector
GW_cassette_P2	CATCACCACTTTGTACAAGAAAGCTG	Construction of pUGW_sfGFP_HSPT vector
orf137_CR-RT	TAGGCGATCTCAGGTTGACCG	cDNA synthesis of circularized RNA
orf137_CR-RT_Fw1	AGGATTAACGCAAATGTATATTGATC	1st PCR in the cR-RT-PCR experiment
orf137_CR-RT_Rv1	GGTCGAGTTAAGGTGAAGACTG	1st PCR in the cR-RT-PCR experiment
orf137_CR-RT_Fw2	GGAGTAACGAGTAAGTACTTTTCTT	2nd PCR in the cR-RT-PCR experiment
orf137_CR-RT_Rv2	ATCACGGAACACTTTCTATATCTAAG	2nd PCR in the cR-RT-PCR experiment

†Nucleotide shown in small letter missmatch for dCAPS marker
